# Mr. Warren's Case of Fungous, after Fracture

**Published:** 1806-08

**Authors:** S. Warren

**Affiliations:** Milverton, Somerset


### To Dr. BATTY.

Sir,

MOST surgeons have experienced great difficulty in subduing those fungous excrescences which arise from the cerebrum, or its membranes, after the operation of trepanning. Such morbid productions are always dangerous, and I may add from the experience of gentlemen of the first professional eminence, they generally prove fatal. The remedies proposed in common treatises on surgery are insufficient: they are not grounded on any principles, nor detailed with that accuracy which the importance of the subject demands. The structure of such tumours has not yet been demonstrated, nor are the circumstances which tend to produce them known. Morgagni has thrown no light on the subject, and by Dr. Baillie it has been only mentioned superficially. The annexed case, in which an excrescence from the cerebrum, of unusual magnitude, was successfully treated, may therefore, perhaps, be deemed worthy a place in your valuable Journal. I have no theory to offer, nor any new remedy to propose; but every state of the case is particularly described, and I wish to invite those gentlemen who have opportunity, to investigate a subject which has hitherto received so little illustration from medical science.

I am, Sir, &c.

S. WARREN.

*Milverton, Somerset, March* 26, 1806.

---

On the 8th of August, 1805, Mr. John Butter, a farmer, about 23 years of age, of a sanguine temperament, was kicked by a horse on the superior and posterior part of the right parietal bone. My assistance was immediately required. I found the scalp much bruised and lacerated, and a portion of it, as large as a crown piece, struck off. The pericranium was detached, and a fracture with slight depression of the scull was perceptible near the foramen parietale. The patient was totally deprived of sense, the pupils of his eyes were dilated, he breathed hard, and exhibited other symptoms indicative of compression of the brain. The circumstances of the case were communicated to his friends, and the necessity of applying the trephine was strongly urged. But their great dread of an operation, and the possibility that the symptoms might arise from concussion (the external appearance of depression being slight), induced me to make some previous trial of the efficacy of bleeding and evacuants. I therefore took away 12 ounces of blood, and ordered a purging dose of calomel, and a cathartic enema. On the following day, the evacuations having been insufficient, and the pulse being hard and oppressed, those remedies were repeated. By this treatment, on the 10th, some remission of the symptoms was obtained; but on the morning of the 11th, the coma, stertor, and sickness recurred with so much violence, that I determined to postpone the operation no longer. I removed the whole of the depressed part of the skull, by including it within the circumference of the trephine; and on examining the piece, I observed that a splinter of the inner table, projecting one fourth of an inch from its internal superficies, had pierced the dura mater. No hæmorrhage attended the operation, nor was any portion of the cerebrum lost. The dura mater appeared of a dark purple colour, and the wound in it made by the splinter of bone, was a narrow slit measuring about two-thirds of an inch in length. Simple dressings * were applied to the wound, and a saline mixture with the pulvis antimonialis was prescribed. Strict abstinence had been enjoined from the day on which the injury was received.

On the 12th, I found no alleviation of the symptoms. The patient's pulse still continuing hard and oppressed, he was again bled, and a laxative draught was prescribed.

13th, A favourable change had taken place. The patient's bowels had been copiously evacuated; his pulse was more free; the coma was so much relieved that he began to recognize those who attended him; the iris was again sensible

* White wax ointment thinly spread on lint.

sensible to the stimulus of light; and towards the evening, he uttered a few words, but too indistinctly to be understood. His bowels were in a proper state. The antimonial mixture was repeated.

14th, The patient had slept much during the night, without starting or alarm; he was now able to answer collectedly many questions. His skin was moist and his bowels regular. The wound began to secrete pus, but I observed that the dura mater looked unusually white, and protruded so as nearly to fill up the space left by the removal of the piece of bone. Simple dressings were continued.

15th, No perceptible change.

16th, At two o'clock in the morning a messenger informed me that my patient was materially worse. I found him restless, at times delirious, and complaining of a violent shooting pain in his head, his pulse beating 120 strokes in a minute, his skin hot and dry. Finding that his bowels had not been evacuated during the preceding day, I ordered an enema and a cathartic draught. These soon operated efficaciously: the pain in the head ceased, and the pulse was lowered. The appearance of the wound was not in any degree changed.

17th, The symptoms continued favourable. The patient conversed coherently, and could recollect the circumstances which immediately preceded the injury. His bowels were moderately relaxed. The protruded dura mater completely filled the perforation in the scull made by the trephine; it had entirely lost its silvery tendinous appearance, and looked dull and pale. The wound on the scalp was in a healthy state.

Until the 26th, no particular alteration occurred. On that day I observed, that the dura mater had risen above the external surface of the skull, and a slight pulsation was perceptible beneath. The same dressings were applied, and a strict observance of abstemious diet was enjoined. The general health of the patient gradually returned, but the tumour continued to enlarge. The dura mater began to slough, and fungous granulations from the cerebrum grew rapidly. I first endeavoured to check this excrescence by compression, and the application of argentum nitratum: but the slightest pressure induced dimness of sight and vertigo; I therefore discontinued it, using the caustic only. After a week had elapsed, the disease still increasing, I folded a piece of paste board between a linen cloth, and bound it on the tumour, augmenting the pressure by small degrees. No unfavourable symptom supervened pervened, and I hoped by this means to prevent further protrusion, and that caustics would destroy the fungus already formed; its size, for a time, was evidently diminished, and the pulsation in it became less perceptible. During the month of October, some small pieces of bone were exfoliated from the border of the perforation.

In the beginning of November, the tumour suddenly increased to twice its former dimensions; and finding my endeavours to repress it unavailing, I determined to remove it by ligature. In about four days the whole of it sloughed off, and I attempted to prevent its reproduction by a bandage and pressure. But the granulations now sprung up so rapidly, that in three days a tumour was protruded as large as a turkey's egg, notwithstanding that caustics, and the greatest pressure consistent with safety, had been employed. The patient frequently complained of great pain in his head, he became feeble and low spirited, and at intervals his memory failed. It therefore became necessary to adopt some new plan. I procured two thin plates of silver, of a semi lunar shape, and applied their concave edges laterally to the base of the tumour, drawing them gradually closer to each other by strips of sticking plaister. These plates were daily removed, and argentum nitratum was applied to the line formed round the tumour by their pressure. By this mode the diseased mass was slowly detached, and in the course of fifteen days it was entirely removed. I then covered the perforation in the skull with a circular plate of silver, and retained it securely in its place by pieces of sticking plaister. This plate I was at first obliged to remove daily, in consequence of a considerable secretion of pus; but when the discharge ceased, I found great advantage in allowing the plate to remain unmoved for many days. A few fungous granulations sometimes appeared beneath the plate, but they were destroyed by caustic. In about three weeks the morbid disposition of the wound seemed totally subdued. The plate was removed, and a bandage with a linen compress substituted. The scalp gradually closed and formed a firm cicatrix, but the process of ossification has not yet taken place. The patient is in full possession of his former health and strength, his spirits are good, and his mental faculties unimpaired.

